# The relationship between interpersonal sensitivity and short video addiction among college students: the chain mediation effect of loneliness and fear of missing out

**DOI:** 10.3389/fpsyg.2026.1736377

**Published:** 2026-04-15

**Authors:** Zheng Zhang, Jiajie Gu, Kunbo Wu, Liuping Hou, Gaofeng Zha, Lijie Yuan, Min Xu

**Affiliations:** School of Urban Construction, Changzhou University, Changzhou, China

**Keywords:** college students, fear of missing out, interpersonal sensitivity, loneliness, short video addiction

## Abstract

**Background:**

Short video platforms have rapidly gained popularity among college students, leading to issues of addictive use. Existing research has shown a significant association between interpersonal sensitivity and short video addiction, but the underlying psychological mechanisms remain unclear. This study aims to explore the mediating role of loneliness and fear of missing out in the relationship between interpersonal sensitivity and short video addiction.

**Methods:**

A questionnaire survey method was used to select 465 college students in mainland China. Scales measuring interpersonal sensitivity, loneliness, fear of missing out, and short video addiction were employed for measurement, and mediation analysis was conducted to test the causal pathways.

**Results:**

Interpersonal sensitivity is significantly positively correlated with short video addiction (*p* < 0.01). Loneliness and fear of missing out are both significantly positively correlated with interpersonal sensitivity and short video addiction (*p* < 0.01). Loneliness and fear of missing out both play a significant mediating role in the relationship between interpersonal sensitivity and short video addiction. Loneliness and fear of missing out exhibit a significant chain mediating effect.

**Conclusion:**

Interpersonal sensitivity not only directly affects college students’ addiction to short videos, but also indirectly increases the risk of addiction by exacerbating loneliness and heightening the fear of missing out. Interventions targeting individuals with high interpersonal sensitivity should focus on both loneliness and fear of missing out management to break the vicious cycle between psychology and behavior and reduce the incidence of short video addiction.

## Introduction

With the advent of the self-media era, emerging social media platforms have risen rapidly. Short videos, with their immediacy, fragmentation, and strong interactivity, have quickly become an important medium for contemporary college students’ daily entertainment and social interaction. According to statistics, by December 2024, the number of short video users in China had reached 1.091 billion, with college students accounting for over 40% of the total (Yuan and Wang, 2024). Short videos have not only transformed the way information is disseminated but have also profoundly influenced young people’s lifestyle habits and social interaction patterns. However, as the use of short videos has become widespread, the chronic addiction resulting from their frequent use has raised concerns about addiction issues, manifested as excessive dependence, withdrawal symptoms, and functional impairment (Christian et al., 2019). Research indicates that short video addiction among college students is significantly associated with academic burnout, anxiety, and depression (Gu et al., 2023), suggesting that short video use may have negative impacts on mental health.

Although existing research has explored the association between short video addiction and various psychological issues, in-depth investigations into the mechanisms underlying the formation of short video addiction and psychological issues remain insufficient, particularly when considering individual differences. Interpersonal sensitivity, as a core personality trait reflecting excessive vigilance and reactivity to social cues and evaluations, is a crucial individual difference factor that may predispose individuals to maladaptive coping strategies in digital environments (Zheng et al., 2025). Interpersonal sensitivity, as a personality trait, reflects an individual’s abnormal sensitivity to others’ evaluations in social contexts (Dong et al., 2024). This trait may exacerbate an individual’s vulnerability when facing negative social comparisons and further impact their mental health status. However, the specific pathways through which this sensitivity translates into compulsive short video use are not well understood. We posit that loneliness and fear of missing out (FOMO) are two key psychological states that may serve as critical mediators in this process, bridging stable personality traits (interpersonal sensitivity) and specific addictive behaviors (short video addiction).

Loneliness, defined as the perceived deficit in one’s social relationships, is a common experience among college students and is often heightened in individuals with high interpersonal sensitivity due to social avoidance and misinterpretation of social cues. According to social needs theory, unmet belongingness needs can drive compensatory behaviors (Holte et al., 2024). Fear of missing out (FOMO), characterized by a pervasive anxiety about missing rewarding experiences others might be having, is particularly salient in the context of social media and short video platforms, which constantly broadcast curated snippets of others’ lives (Türk-Kurtça, 2026). For individuals highly sensitive to social information, this environment may fuel FOMO. We hypothesize that interpersonal sensitivity predisposes individuals to experience greater loneliness (due to strained real-world interactions) and heightened FOMO (due to hyper-vigilance to social comparisons online). In turn, both loneliness and FOMO may drive excessive short video use as a maladaptive coping mechanism—loneliness for seeking connection and FOMO for staying continuously informed. Furthermore, these two states may not be independent; loneliness may exacerbate FOMO as individuals desperately seek social information to compensate for their isolation, forming a vicious cycle that amplifies addiction risk (Wang et al., 2024). Existing research is limited in its exploration of how interpersonal sensitivity influences short video addiction through specific psychological processes. Therefore, clarifying these complex interactive relationships is crucial for comprehensively understanding the psychological mechanisms underlying short video addiction.

This study aims to investigate the relationship between interpersonal sensitivity and short video addiction among college students, with a particular focus on the chain-mediated role of loneliness and fear of missing out between the two. Through this study, we hope to gain a deeper understanding of the psychological principles underlying short video addiction and provide scientific evidence for developing effective intervention strategies to improve college students’ mental health and quality of life. Additionally, the findings of this study may offer valuable insights for educators and policymakers to promote the creation of a healthier digital environment.

Interpersonal sensitivity is an important concept in personality psychology, referring to an individual’s tendency to overly perceive and react to others’ emotions, attitudes, and social cues (Lloyd and Hugenberg, 2021). Its characteristics include fear of negative evaluations, excessive reflection on social situations, and catastrophic expectations of outcomes in relational conflicts (Georgiades et al., 2023). From a cognitive-behavioral perspective, individuals with high interpersonal sensitivity often exhibit significant attentional biases, tending to prioritize processing threatening signals in social environments. This may lead to excessive emotional arousal and inappropriate response behaviors (Falco et al., 2024) This cognitive pattern may trigger real-life social avoidance, leading college students to increasingly rely on virtual interactions on short video platforms to seek a sense of security (Wang et al., 2025). Based on a longitudinal study of college students, individuals with higher levels of interpersonal sensitivity tend to alleviate social anxiety through immediate feedback such as likes and comments on short videos. However, this alternative sense of fulfillment may gradually evolve into addictive behavior (Nong et al., 2023; Ye et al., 2023b). Therefore, we propose hypothesis H1.

*H1:* Interpersonal sensitivity can positively predict short video addiction.

Loneliness refers to the emotional isolation and lack of connection an individual perceives within their social network (Mund and Johnson, 2021). In recent years, it has become increasingly prevalent among college students. According to social needs theory (Steverink et al., 2020), when individuals are unable to obtain emotional support and a sense of belonging in real-life social interactions, they may experience a persistent state of psychological alienation. Individuals with high interpersonal sensitivity, due to their excessive focus on others’ evaluations and tendencies toward social avoidance (Hall et al., 2009), often experience significantly impaired social interaction quality. On one hand, highly socially sensitive individuals often overinterpret social feedback (e.g., misinterpreting others’ brief silences as rejection), leading to cognitive rumination and further exacerbating feelings of loneliness (Primack et al., 2017); on the other hand, chronic loneliness may enhance an individual’s social vigilance, creating a vicious cycle of “sensitivity-loneliness-greater sensitivity” (Cacioppo et al., 2014). Based on the compensatory network usage theory (Tsao, 1996), individuals feeling lonely may seek alternative social experiences through short video platforms, as the platform’s fragmented information and high-frequency interactive feedback precisely meet individuals’ needs for immediate emotional release (Zhao and Kou, 2024). Short video platforms use precise algorithms to push personalized content, creating simulated intimate relationships for lonely individuals. However, such digital connections inherently cannot provide genuine interpersonal support. Instead, through the “pleasure peak-emptiness trough” neural regulation mechanism, they may induce users to develop dependent behaviors, ultimately trapping individuals in a vicious cycle of heightened emotional sensitivity, deepened loneliness, and increased usage intensity (Hernández et al., 2024; Ye et al., 2024b; Zhang et al., 2025). Accordingly, this study proposes Hypothesis H2.

*H2:* Interpersonal sensitivity indirectly enhances short video addiction by exacerbating loneliness, meaning that loneliness plays a mediating role in this relationship.

Fear of Missing Out (FOMO) is a typical psychological phenomenon that has emerged in the digital age, referring to a persistent state of anxiety individuals experience due to concerns about missing out on others’ experiences or social opportunities (Kaddouhah, 2024). Its core characteristics include excessive focus on others’ activities, an uncontrollable urge to seek information, and a tendency toward “always-on” behavior. Social media environments particularly amplify this psychological phenomenon, as platform designs continuously reinforce users’ demand for real-time insights into others’ lives (Servidio et al., 2024). Individuals with higher interpersonal sensitivity, who are more attuned to social cues, are typically more prone to falling into the psychological trap of social comparison. They tend to assess their own social value and sense of belonging by constantly observing others’ life statuses and social activities (Uzum et al., 2022), thereby intensifying their focus on social information and increasing the severity of FOMO. The infinite refresh mechanism and personalized content recommendation features of short video platforms provide an immediate emotional outlet to alleviate this anxiety. Users gain temporary psychological satisfaction by continuously swiping the screen and browsing new content, thereby forming a habitual or even dependent usage pattern (Yin et al., 2023). Research indicates that individuals with higher levels of FOMO often exhibit stronger tendencies toward short video usage, and their usage behavior also exhibits distinct ritualistic characteristics, meaning they experience intense urges to use the platform in specific situations and alleviate inner anxiety by watching short videos (Wegmann et al., 2017; Ye et al., 2023a; Ye et al., 2025a; Ye et al., 2025b). Based on the above analysis, this paper proposes Hypothesis H3.

*H3*: Interpersonal sensitivity indirectly promotes short video addiction by inducing FOMO, specifically by mediating the relationship between interpersonal sensitivity and short video addiction.

Previous sections have separately examined the independent mediating roles of loneliness and fear of missing out in the relationship between interpersonal sensitivity and short video addiction. However, these two factors are not mutually exclusive in actual psychological mechanisms but exhibit significant interactive effects. According to emotional compensation theory, when individuals experience loneliness, their psychological sense of security is weakened, leading to excessive focus on social information to restore a sense of control (Hayes, 2013). This compensatory need may evolve into FOMO, creating a vicious cycle where loneliness leads to information craving, which in turn exacerbates loneliness (Bonfanti et al., 2023). For example, Wolniewicz et al. (2017) found that loneliness indirectly promotes social media dependency by intensifying FOMO, a phenomenon particularly pronounced among college students. During short video use, lonely individuals may seek a sense of presence through frequent refreshing, yet this behavior actually widens the gap from real-world social interactions and further exacerbates short video addiction (Ye et al., 2024a; Ye et al., 2022). This bidirectional mechanism suggests that loneliness and fear of missing out may form a chained mediating pathway: interpersonal sensitivity → loneliness → fear of missing out → short video addiction. Based on this, Hypothesis H4 is proposed.

*H4:* Interpersonal sensitivity positively influences short video addiction through the chain-mediated effects of loneliness and fear of missing out.

**FIGURE 1:**
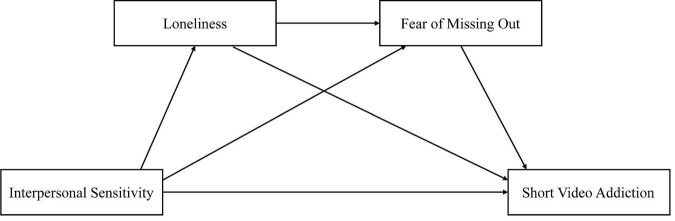
The chain mediation model.

In summary, this study aims to explore the relationship between interpersonal sensitivity, loneliness, fear of missing out, and short video addiction, as well as the mediating role of loneliness and fear of missing out between interpersonal sensitivity and short video addiction. Ultimately, it seeks to reveal the generative mechanism and psychological mechanism by which interpersonal sensitivity influences short video addiction. The chain mediation model is shown in Figure 1.

## Materials and method

### Participants

This study employs a cross-sectional design to examine the relationship between interpersonal sensitivity and short video addiction, with loneliness and fear of missing out serving as mediating variables. The research was conducted at a university in Changzhou City, Jiangsu Province, China. Ethical approval was obtained from the Ethics Review Committee of the School of Urban Construction at Changzhou University to ensure compliance with relevant research guidelines. The sampling method adopted in this study is convenience sampling, which is a non-probability sampling technique. Sample selection is primarily based on accessibility or convenience. To encourage participants to engage in the study, individuals who are most easily accessible are selected as samples.

This study employed an online survey platform^1^ to distribute the questionnaires. After obtaining approval from the relevant college counselors and class advisors of the university, the questionnaire link and QR code were randomly disseminated to all undergraduate students through online social channels such as class WeChat and QQ groups. The average completion time for the questionnaire was approximately 3–5 min. To improve the response rate, two reminder messages were sent via class advisors on the third and seventh days following the initial distribution. A total of 520 questionnaires were distributed, with 516 returned. After thorough screening to exclude 51 incomplete questionnaires, 465 valid questionnaires were identified, yielding a response rate of 90.12%. The respondents were undergraduate students from the first to the fourth year with an average age of approximately 20 years. Among these, 324 were male (69.68%) and 141 were female (30.32%). Participants were categorized by academic year: 99 first-year students (21.29%), 108 second-year students (23.22%), 145 third-year students (31.18%), and 113 fourth-year students (24.31%).

Before participants took the questionnaire, we explained the purpose and significance of the survey in detail and assured them that the data collected would only be used for research purposes. Throughout the study, participants were completely voluntary and anonymous. Participants also had the right to withdraw from the study at any time.

### Research tools

#### Interpersonal sensitivity scale

This study used the interpersonal sensitivity measurement items in the SCL-90 compiled by Derogatis and Unger (2010) and revised by Wang (1984) to measure interpersonal sensitivity. The scale consists of nine items and uses a five-point rating scale, with “1” representing “none” and “5” representing “severe.” In this study, its Cronbach’s alpha coefficient was 0.914.

#### Loneliness scale

To more accurately capture the current state of loneliness among college students, the UCLA Loneliness Scale, originally developed by Russell et al. (1980) and later revised by Wang et al. (1999), was adopted in this study. This scale consists of 20 items and uses a 4-point scoring system, where “1” represents “never” and “4” represents “always.” Some of the items are reverse-scored. In this study, the Cronbach’s alpha coefficient was 0.939.

#### Fear of missing out scale

The Chinese version of the FOMOS scale, developed by Przybylski et al. (2013) and revised by Qi et al. (2019), was used. The questionnaire is divided into two dimensions: fear of missing information and fear of missing situations, each with four items. Scoring is on a 5-point scale (“1 = completely disagree” to “5 = completely agree”), with higher total scores indicating higher levels of fear of missing out. In this study, the Cronbach’s alpha coefficient was 0.925.

#### Short video addiction scale

The College Students’ Short Video Addiction Scale developed by Qin (2020) was used. The scale consists of four dimensions: loss of control, withdrawal, avoidance, and inefficiency, with a total of 14 items scored on a 5-point scale (“1 = completely disagree” to “5 = completely agree”). In this study, the Cronbach’s alpha coefficient was 0.927.

#### Data analyses

Descriptive statistics and Pearson correlation analysis were performed using SPSS 27. The chain mediation model was tested using the PROCESS macro for SPSS (Model 6). The mediating effects in the mediation analysis were examined using the Bootstrap method, with 5,000 resamples to generate 99% confidence intervals (CIs). The mediating effect was considered statistically significant if the 99% confidence interval did not contain zero (Kline, 2018).

## Results

### Common method bias test

In this study, when analyzing data using structural equation modeling, common method bias (CMB) was systematically examined. Exploratory factor analysis (EFA) was conducted on all measurement items using Harman’s single-factor test method. The results showed that no factor rotation yielded four factors with eigenvalues greater than 1, with the largest factor explaining 37.53% of the variance, which did not exceed the critical threshold of 40% (Podsakoff et al., 2003), indicating that while the data exhibit a certain degree of common method bias, it does not yet pose a serious threat to the discriminant validity of the model construction.

### Descriptive statistics and correlation analysis

Table 1 shows the results of descriptive statistics and correlation analysis conducted using SPSS 25.0: interpersonal sensitivity, loneliness, fear of missing out, and short video addiction were all significantly positively correlated (*r* = 0.616–0.708, *p* < 0.01), which meets the statistical requirements for subsequent mediation effect analysis of loneliness and fear of missing out (Baojuan et al., 2013). Additionally, to determine whether other factors needed to be controlled in subsequent analyses, basic variables such as gender, age, major, and whether the individual was an only child were correlated with the four variables under study. The results show that gender is significantly positively correlated with loneliness, while grade level is significantly negatively correlated with interpersonal sensitivity and short-video addiction. Therefore, gender and grade level were included as control variables in subsequent regression analyses.

**TABLE 1:** Correlation analysis between interpersonal sensitivity, loneliness, fear of missing out, and short video addiction.

Variable	M	SD	1	2	3	4	5	6
1. Gender	1.303	0.460	–	–	–	–	–	–
2. Grade	2.585	1.076	–0.076					
3. Interpersonal sensitivity	2.209	0.991	0.080	–0.091*				
4. Loneliness	2.104	0.760	0.092*	–0.064	0.708**			
5. Fear of missing out	2.234	1.097	0.041	–0.006	0.666**	0.688**		
6. Short video addiction	2.385	1.040	0.078	–0.111*	0.616**	0.643**	0.690**	

M, mean; SD, standard deviation. *At the 0.05 level, the correlation is significant. **At the 0.01 level, the correlation is significant.

### The mediating role of loneliness and fear of missing out between interpersonal sensitivity and short video addiction

**FIGURE 2:**
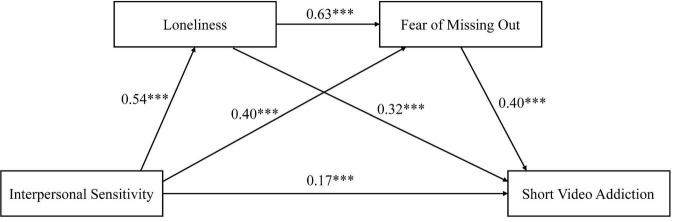
A mediating role model of interpersonal sensitivity in short video addiction. ***Indicates that the *p*-value is less than 0.001, meaning that the results are highly statistically significant.

According to Ye and Wen (Baojuan et al., 2013) on the application of mediation effects, combined with Hayes’ method of using the PROCESS macro program in SPSS to test multiple mediation models, model 6 in PROCESS was used to conduct a multiple hierarchical regression analysis (Hayes, 2013). Grade and gender were used as control variables, interpersonal sensitivity as the independent variable, loneliness and fear of missing out as mediating variables, and short video addiction as the dependent variable for analysis. The results (see Figure 2 and Table 2) indicate that interpersonal sensitivity positively predicts loneliness (γ = 0.54, *t* = 21.35, *p* < 0.001), and it significantly predicts fear of missing out (γ = 0.40, *t* = 8.13, *p* < 0.001). Loneliness positively predicts fear of missing out (γ = 0.63, *t* = 9.70, *p* < 0.001) and interpersonal sensitivity, loneliness, and fear of missing out all positively predict short video addiction (γ = 0.17, *t* = 3.45, *p* < 0.001; γ = 0.32, *t* = 4.72, *p* < 0.001; γ = 0.40, *t* = 9.11, *p* < 0.001).

**TABLE 2:** Regression analysis of variable relationships in a chain mediation model.

Equation of regression		Overall fit index			Significance of regression coefficient		
Result variable	Variable of prediction	*R*	*R* ^2^	*F*	β	*t*	*p*
LON	IPS	0.591	0.350	61.833	0.315	14.626	0.000***
	Gender				0.111	2.428	0.016*
	Grade				–0.063	–3.204	0.001**
	Only child				0.002	0.052	0.958
FOMO	IPS	0.739	0.547	110.659	0.465	12.310	0.000 ***
	LON				0.629	9.311	0.000 ***
	Gender				–0.009	–0.131	0.896
	Grade				0.005	0.192	0.848
	Only child				–0.045	–0.729	0.466
SVAD	IPS	0.764	0.584	107.274	0.208	4.820	0.000***
	LON				0.192	2.634	0.009**
	FOMO				0.546	11.802	0.000***
	Gender				0.032	0.488	0.626
	Grade				–0.033	–1.152	0.250
	Only child				0.041	0.680	0.497

*p < 0.05, **p < 0.01, ***p < 0.001.

The nonparametric percentile Bootstrap method with bias correction was used to test the mediating effect. Repeated sampling was performed 5,000 times to calculate the 95% confidence interval. If the confidence interval did not include the value 0, it indicated statistical significance. The results are shown in Table 3: The direct effect analysis revealed that for each one-standard-deviation increase in interpersonal sensitivity, the severity of short video addiction directly increased by 0.17 units (95% CI = [0.07, 0.27]), accounting for 26.56% of the total effect (0.64). This result validates the independent predictive role of interpersonal sensitivity on short video addiction (Kardefelt-Winther, 2014), suggesting that individuals’ excessive sensitivity to social cues may directly drive them to use short videos to escape real-world pressures or seek immediate emotional comfort.

**TABLE 3:** The mediating effect of interpersonal sensitivity on short video addiction.

Effect	Path relationship	Effect value	Standard error	95%CI	Magnitude of effect
Direct effect	Interpersonal sensitivity → Short video addiction	0.17	0.05	[0.07, 0.27]	26.56%
Mediation effect	Interpersonal sensitivity → Loneliness → Short video addiction	0.17	0.05	[0.08, 0.28]	26.56%
	Interpersonal sensitivity → Fear of missing out → Short video addiction	0.16	0.04	[0.09, 0.24]	25.00%
	Interpersonal sensitivity → Loneliness → Fear of missing out → Short video addiction	0.14	0.03	[0.09, 0.19]	21.88%
Total mediation effect		0.47	0.05	[0.38, 0.56]	73.44%
Total effect		0.64	0.04	[0.56, 0.72]	

Further mediation analysis revealed three significant pathways: (1) the mediating effect of loneliness (interpersonal sensitivity → loneliness → short video addiction). The effect size of this pathway was 0.17 [95% CI = (0.08, 0.28)], accounting for 26.56% of the total effect. Individuals with high interpersonal sensitivity often experience social anxiety due to overinterpreting others’ behaviors, which in turn leads to loneliness (Fang et al., 2020). The immersive experience of short videos can temporarily fill emotional voids, leading to behavioral dependence. (2) The mediating path of fear of missing out (interpersonal sensitivity → fear of missing out → short video addiction) has an effect size of 0.16 [95% CI = (0.09, 0.24)], accounting for 25.00% of the total effect. Interpersonally sensitive individuals tend to overly focus on others’ activities and fear missing out on social information (Przybylski et al., 2013). This psychological pressure drives them to frequently refresh short video content to alleviate anxiety. (3) The chain mediation path (interpersonal sensitivity → loneliness → fear of missing out → short video addiction) had an effect size of 0.14 [95% CI = (0.09, 0.19)], accounting for 21.88%. This suggests that loneliness may exacerbate fear of missing out, further leading to addictive behavior. Thus, the hypothesis of this study was validated.

## Discussion

### The impact of interpersonal sensitivity on short video addiction

The regression model in the study showed that interpersonal sensitivity significantly and positively predicted short video addiction, meaning that college students with high interpersonal sensitivity exhibited more pronounced short video addiction. Interpersonal sensitivity, as a personality trait characterized by excessive awareness and reaction to social cues, may directly contribute to short video addiction through cognitive-behavioral mechanisms. Highly socially sensitive individuals tend to prioritize processing threatening social signals in their environment (such as negative evaluations or exclusionary cues from others). This attentional bias can trigger emotional arousal and social anxiety (Leigh and Clark, 2018), prompting individuals to substitute virtual interactions on short video platforms for real-world social interactions. The immediate feedback features of short videos (such as likes and comments) can temporarily alleviate interpersonal sensitivity individuals’ feelings of self-doubt, leading to behavioral dependence (Zhang et al., 2024). In this study, the direct effect of interpersonal sensitivity on short video addiction accounted for 26.56% of the total effect, indicating that a substantial portion of its influence operates independently of loneliness and FoMO. When real-world social relationships fail to provide sufficient psychological support and a sense of belonging, individuals tend to utilize virtual interactions to compensate for this deficiency, thereby achieving emotional balance and support (Kardefelt-Winther, 2014).However, the relationship between interpersonal sensitivity and media use may not be uniformly straightforward. Some studies suggest that individuals with high social anxiety—a construct closely related to interpersonal sensitivity—may actually avoid certain types of socially exposed online activities, leading to more passive or solitary media use rather than interactive engagement (Hutchins et al., 2021). This indicates that the motivational drivers and behavioral outcomes linked to sensitivity can be complex and platform-dependent.

### The mediating effect of loneliness

The present study suggests that loneliness serves as a mediating variable between interpersonal sensitivity and short video addiction, with interpersonal sensitivity positively predicting short video addiction. According to the Social Needs Theory (Doyal and Gough, 1984), individuals with high interpersonal sensitivity struggle to establish stable real-world connections due to social avoidance and cognitive rumination, leading to heightened feelings of loneliness (Primack et al., 2017). Short video platforms use algorithmic recommendation technology to deliver personalized content, providing immediate emotional comfort to lonely individuals. However, their fragmented interactions cannot replace genuine social support and may instead exacerbate addiction cycles through neural adaptation mechanisms (Daria and Mark, 2017). This study indicates that loneliness not only stems from interpersonal sensitivity but also increases the risk of addiction through compensatory use behavior, meaning that loneliness can positively predict short video addiction to a certain extent. Conversely, it is important to note that some longitudinal research presents a different perspective, where loneliness is shown to be a consequence, rather than a primary antecedent, of problematic social media use (O’Day and Heimberg, 2021). This bidirectional potential suggests that while our model posits loneliness as a mediator, the compensatory use of short-form videos might, over time, deepen feelings of social disconnection for some individuals, creating a feedback loop.

### The mediating effect of fear of missing out

Fear of missing out (FOMO) serves as a typical anxiety in the digital age and is another important mediating variable influencing short video addiction through interpersonal sensitivity. Individuals with high interpersonal sensitivity tend to be overly vigilant toward social cues, which can trigger a tendency toward social comparison (Vogel et al., 2015). This leads them to continuously monitor others’ activities to assess their own social value, thereby exacerbating FOMO (Przybylski et al., 2013). The infinite refresh mechanism and trending recommendations feature of short video platforms provide an outlet for alleviating such anxiety, but high-frequency use can exacerbate ritualistic behaviors, such as compulsively scrolling through videos before bed, creating a vicious cycle of “anxiety-use-more anxiety” (Wegmann et al., 2017). It is critical to integrate contrasting findings, such as those indicating that FOMO’s relationship with addiction might be moderated by factors like self-esteem or platform type. For instance, some users high in FOMO may engage in more active, communication-based social media use rather than passive consumption of short-form videos (Seidman et al., 2025). This highlights that the pathway from interpersonal sensitivity through FOMO to addiction may be specific to the immersive, rapidly shifting content format characteristic of short-form video platforms.

### The chain mediation effect of loneliness and fear of missing out

Research has found that loneliness can positively predict fear of missing out to a certain extent, and fear of missing out can positively predict short video addiction. According to the emotional compensation theory, loneliness weakens an individual’s psychological sense of security, driving them to frequently seek social information to rebuild a sense of control (Peng et al., 2025). However, this compensatory behavior actually exacerbates FOMO, which in turn exacerbates short video addiction. That is, loneliness and FOMO can play a chain-like mediating role between interpersonal sensitivity and short video addiction. Nevertheless, the proposed sequential relationship (loneliness → FOMO) is not universally supported. Alternative models exist where FOMO is posited as a precursor to feelings of loneliness, as the constant exposure to others’ curated highlights may foster perceptions of social inferiority and isolation (Čifči and Kumcağız, 2023). This debate underscores the complexity of these psychological states and suggests that the chain mediation observed in our study might represent one of several potential dynamic pathways.

### Research value

This study holds significant innovative value both theoretically and practically. It provides a substantial theoretical contribution by delineating the psychological mechanism through which interpersonal sensitivity leads to short - video addiction, a distinct and prevalent form of digital dependency in the mobile internet era. While prior research has established associations between personality traits and generalized internet addiction (Wang et al., 2015), this work deepens understanding by uncovering the chained mediation pathway of loneliness and fear of missing out (FOMO). It moves beyond mere correlation to reveal the sequential emotional - cognitive process that underlies platform-specific addictive behavior. The findings integrate and extend several theoretical frameworks, including interpersonal deficiency theory, compensatory internet use theory, and cognitive-behavioral models. They demonstrate how loneliness and FOMO work in tandem to transform a stable personality trait into compulsive short-video use. This process-oriented perspective not only enriches the cognitive-behavioral model with socio-emotional mediators but also highlights the temporal dynamics between emotional isolation and cognitive preoccupation in digital environments. By unpacking the “black box” between interpersonal sensitivity and behavioral addiction, the study offers a refined theoretical lens for understanding how technology-mediated coping evolves. It thereby provides a foundation for more nuanced research on contemporary digital pathologies.

This study provides clear practical implications for mental health education in higher education, the regulation of short-form video platforms, and student self-management. It was found that students with higher interpersonal sensitivity are more inclined to use short-form video platforms as a “digital refuge” to alleviate social anxiety, with loneliness and fear of missing out (FoMO) playing a chain-mediating role in this process. Based on these findings, it is suggested that educators and mental health practitioners develop dual-track interventions for interpersonally sensitive students. First, real-world social skills can be enhanced through group counseling and social skills training to alleviate feelings of loneliness. Second, cognitive-behavioral interventions can be implemented to help students recognize and regulate FoMO, thereby reducing compulsive video-watching behaviors driven by information-seeking. At the institutional level, it is recommended that media literacy education be strengthened to promote healthy short-form video usage habits, while more offline interaction opportunities should be created through curricula and activities to mitigate virtual dependency. For short-form video platforms, it is advised that recommendation algorithms be optimized, and features such as anti-addiction reminders and usage duration feedback be introduced to avoid excessive stimulation of users’ FoMO and prolonged usage behaviors. Furthermore, families and society should jointly address the psychological needs of highly sensitive individuals, fostering a balanced online-offline social support system. These measures may help break the vicious cycle of “sensitivity–loneliness–FoMO–addiction” and promote a healthier digital lifestyle.

### Insufficient research and prospects

Although this study has made some theoretical progress, several limitations remain that require further exploration. First, while the cross-sectional design validated the mediating effect through statistical methods, it cannot rule out the possibility of reverse causality, such as excessive use of short videos potentially reinforcing interpersonal sensitivity traits. Second, the sample was limited to undergraduate students in Jiangsu Province, excluding rural-urban differences and individuals with varying educational backgrounds. Additionally, the collected data showed a significant gender imbalance, which may affect the generalizability of the study’s conclusions. Third, the study did not examine the moderating effects of contextual variables such as short video content types and usage scenarios. Existing research suggests that knowledge-based and entertainment-based short videos may have differentiated effects (Huang et al., 2022). Future research could employ longitudinal tracking using experiential sampling methods and incorporate moderating variables such as media content characteristics to more comprehensively elucidate the formation mechanisms of digital addiction.

## Conclusion

This study examined the relationship between loneliness and regulatory emotional self-efficacy, with satisfaction with life and social interaction anxiety serving as mediating variables. The findings suggest that loneliness negatively influences regulatory emotional self-efficacy either directly or indirectly through the mediating effect of satisfaction with life and social interaction anxiety. Notably, satisfaction with life and social interaction anxiety also serves as a sequential mediator, highlighting the complex interplay between cognitive and emotional factors in shaping individual emotional self-regulation. These results underscore the importance of addressing loneliness among college students to enhance emotional self-efficacy. Interventions aimed at improving life satisfaction and reducing social interaction anxiety may be effective strategies for mitigating the negative impact of loneliness on emotional regulation. Future research should explore other sociopsychological variables.

## Data Availability

The raw data supporting the conclusions of this article will be made available by the authors, without undue reservation.

## Footnotes

1
www.wjx.cn
